# The Facies dolorosa and the Conidae

**DOI:** 10.5414/CP202704

**Published:** 2016-07-01

**Authors:** Barry G. Woodcock

**Affiliations:** Johann-Strauss-Strasse, Rödermark, Germany

## Abstract

Editorial

## Editorial

*“The face of the patient, together with the eyes of the physician, constitute the most powerful diagnostic tool”*

This issue of *The International Journal of Clinical Pharmacology and Therapeutics* commemorates the 60^th^ Anniversary of the book “Facies dolorosa” published in an extended and unabridged edition in 1956 by *Dustri* Medical Science Publications [1]. The contents of this unique and highly sought after book, compiled and annotated as a contribution to medical research and teaching by the surgeon and anesthetist Hans Killian, are reviewed appropriately in this commemorative issue by V. Luger and her colleagues at *Dustri* Medical Science Publications. 

Other peer-reviewed contributions, mainly from invited authors, address the clinical pharmacology of pain and pain management. Keynote reviews include Pain Management by Phil Wiffen and Andrew Moore and Neonatal Pain by Karel Allegaert and John van den Anker. The clinical limitations of morphine-related drugs, both in terms of pain relief achievable and safety are well known, but a new treatment strategy, one perhaps of value in the management of intractable neuropathic pain, is emerging based on conopeptides. Conopeptides, or conotoxins, are the active molecular species in the venom of marine gastropods of the genus Conidae. Paradoxically, when conus snail venom enters the body in human stingings it causes excruciating pain and has resulted in many fatalities. The reports on such events have been collected in a database and analyzed here in a report by Alan J. Kohn, a world authority on the zoology and phylogeny of conus gastropods, and in particular on human stingings caused by these snails. The report by Kohn is complemented by a contribution by the late Lady Elizabeth Brown, Edward K.L. Masinde, and Barry G. Woodcock on the pharmacological action of conus venom which is based on research done on location in Tanzania, East Africa. Other contributions include a report by Regina Sit, Wendy Wong, S.W. Law, and Justin C.Y. Wu on Integrative Western and Traditional Chinese Medicine and a Viewpoint and update on the recent French experience with FAAH inhibitors in clinical trials by Christophe Mallet, Claude Dubray, and Christian Dualé. 

Interest in this commemorative issue and submissions of manuscripts on the clinical pharmacology of pain have been substantial. Therefore, a follow-up publication is planned, either as Part II or as a series of single contributions under the rubric “Facies dolorosa” in the regular monthly issues of the journal. 

## Note on the cover image of this issue of the journal 

**Figure 1.**
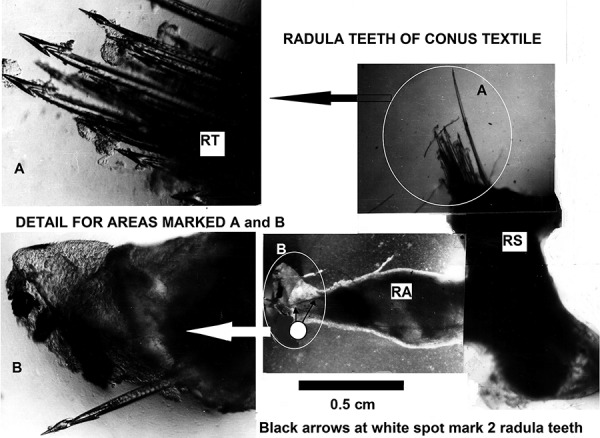
Photomicrographs of the Y-shaped radula sac (RS) from *Conus textile* after dissection exposing ~ 20 radula teeth (RT) in the main part of the organ with their points orientated to the blind end and at least 2 radula teeth in the “ready arm” (RA) ready for immediate deployment. Deployment involves transfer into the pharynx to which the ready arm is connected, loading of the lumen of the “harpoons” with venom from the venom duct in the region of the pharynx (not shown), grasping of the tooth by invaginations of the proboscis, and then impaling the prey with the harpoon held in the proboscis and with the aid of the conspicuous barbs on the radula teeth.

The conopeptide approved for clinical use in the USA, specifically the now synthetically synthesised zirconitide (Prialt^®^), is administered by intrathecal injection beginning at a dose rate of 2.4 µg/day as acetate [2]. The photomicrographs in the cover image with the annotation below illustrate the ironic, even *uncanny, but naturally occurring analogy* of this clinical scenario. In the case of the clinical use of conopeptides the aim of course is to produce pain relief with a therapeutic dose, in the case of conus gastropods, the “deadly harpoon” carries a conopeptide “cocktail” for a quite different purpose. The mechanisms involved in the zoological scenario are described with reference to Figure 1 containing photomicrographs obtained during the investigations of Brown et al. (This issue of the journal [3]). Figure 1 shows part of the venom apparatus of *Conus textile*. This organ is located deep within the body of the animal. The chinitinous harpoons or “radula teeth” used in capturing and killing prey are hollow and fill with venom from the venom duct prior to being thrust by the proboscis into the victim. The radula teeth in this animal, with a shell length of 7 cm, are only 1 – 2 cm long with an external diameter of 0.1 – 0.2 mm. This is an indication of the high pharmacological potency of conopeptides, where a sting can cause blockade of neuronal and neuromuscular function resulting in human fatalities. An excellent raster electron micrograph of a single radula tooth is shown in the contribution by Alan Kohn. However, since photomicrographs showing radula teeth stored in the quiver-like radula sac and “ready arm” of this organ are rarely reported, those shown here will be of didactic interest to clinical pharmacologists not familiar with this area of pharmacology and which, for the reasons mentioned above and others discussed in this issue, is at the cutting edge of clinical research on pain relief. 

## Footnote 

According to Manuel Jimenez Tenorio (Personal communication), an authority on the biology and physiology of Conidae at the Facultad de Ciencias, Universidad de Cádiz, radula teeth are in continuous formation and are stored inside the radula sac, which is attached to the pharynx near the foregut. In the radula sac, all the teeth are held together forming a ribbon and when mature they become loose, detach from the radula ribbon, and move to another section of the sac, the “ready arm” for immediate use. The radula sac and the pharynx have muscles that assist in this purpose. There are very few teeth in the ready arm compared to the rest of the radula sac. Furthermore, the “venom gland” which is a muscular organ at the opposite end of the venom duct, allows the venom formed inside the duct to impregnate the radula tooth after it enters the pharynx, and possibly also assists in the ejection process. See also [4, 5].
